# History taking and response to an adult diarrheal case among community drug retail outlets in Gondar town, north-west Ethiopia: a simulated-client survey

**DOI:** 10.1186/s40545-021-00310-1

**Published:** 2021-03-01

**Authors:** Dawit Wondimsigegn, Berhanemeskel Woldegerima, Asefa Adimasu Taddese

**Affiliations:** 1 Department of Pharmaceutics and Social Pharmacy, School of Pharmacy, College of Medicine and Health Sciences, University of Gondar, Gondar, Ethiopia; 2 Department of Epidemiology and Biostatistics, Institute of Public Health, College of Medicine and Health Sciences, University of Gondar, Gondar, Ethiopia

**Keywords:** Adult diarrhea, Community drug retail outlet, Gondar town, Simulated-client

## Abstract

**Background:**

Self-care is one of the growing tasks of community pharmacy professionals. They are highly engaged in consultations in response to specific drug request (product-based presentation) or symptoms clients describe (symptom-based presentation).

**Purpose:**

This study was aimed at assessing the appropriateness of patient assessment and response to an adult diarrheal case among community drug retail outlets in Gondar town, north-west Ethiopia.

**Methods:**

A descriptive cross-sectional study design based on simulated-client method of visit was conducted from 03 August to 21 September, 2020. An adult female diarrheal case scenario was developed and used to guide data collectors to interact with professionals in a standardized and consistent way. All 60 dispensaries in the town during the data collection were included in the study. A pretested data collection tool was used to record the conversation between simulated clients and providers. The data were analyzed using SPSS version 20.

**Results:**

With regard to patient history, age of the patient, whether diarrhea is watery or bloody and onset and duration of diarrhea were the three most commonly requested questions with 59 (98.3%), 55 (91.7%) and 46 (76.7%), respectively. Past-medical and medication history are enquired in none of the visits. Medication was dispensed in 57 (95%) of the visits and no referral to a health facility was recommended in majority (90%) of cases. The most commonly recommended medications were ciprofloxacin 30 (52.6%) and metronidazole 20 (35.1%). ORS was considered in only 6 (10.5%) dispensaries.

**Conclusion:**

Patient assessment, final decisions and treatment recommendations for an adult diarrheal case are inadequate, irrational and illegal. Educational interventions coupled with incentive mechanisms for cognitive pharmaceutical service and strict regulatory enforcement are needed to reduce the problem.

## Background

Community pharmacy practice involves a multifaceted role and it has undergone a transition from mainly supplying drugs to cognitive pharmaceutical services although the adoption of newly emerging activities differs from country to country [1–5]. One of the growing tasks of pharmacy professionals in community drug retail outlets (CDROs) is facilitation of self-care and guiding consumers to better alternatives. Community pharmacy professionals (CPPs) are uniquely placed to provide support and advice to the general public compared with other health care professionals. The combination of location and accessibility means that most consumers have ready access to a pharmacy where health professional advice is available on demand [6].

Coupled with the increased re-regulation of prescription-only medicines (POMs) to over the counter (OTC) medicines, CPPs are highly engaged in consultations in response to specific drug request (product-based presentation) or symptoms clients describe (symptom-based presentation). The duty of the CPP when responding to symptoms is to make a clear distinction between a minor illness and a more serious condition and hence to ensure that appropriate action is recommended. This may be to advise non-drug treatment, treatment using a suitable non-prescription medicine, or advice to visit a practitioner [7, 8]. In doing so, CPPs need to show professionalism and adhere to national regulations or standards of good pharmacy practice [4, 5, 9]. CPPs are obliged to ensure that the services they are providing to society are of the appropriate quality [10].

However, there are different problems related to the facilitation of self-care. CPPs often fail to adequately assess patients before deciding the type of measures or recommend inappropriate treatment. They showed poor history taking and consultation, incorrect treatment choice, and even consciously dispense POMs without prescription [11–14]. Such practice can seriously affect the care of the patient mainly with medical and economic impacts. One consequence is the risk of resistance. A higher frequency of antimicrobial resistance is reported in communities with high non-prescription antimicrobial use. Safety issues such as adverse drug reactions and masking underlying infectious processes are also associated [15].

In Ethiopia, there were a couple of recent studies that assessed minor illness management including acute childhood diarrhea by CDROs [16–19]. But, no study was conducted on the practice of managing adulthood diarrhea as to our knowledge. So, this study was aimed at assessing the appropriateness of patient assessment and response to an adult diarrheal case among CDROs in Gondar town, north-west Ethiopia. The findings of the study encourage a wider investigation of the quality of pharmaceutical services rendered by CDROs in Ethiopia and devise a mechanism to give standardized patient care.

## Methods

### Study area and period

The study was conducted in Gondar town, Amhara Region, north-west Ethiopia. Based on the 2007 census by the Central Statistical Agency of Ethiopia, the total population of the town is 206, 987 [20]. The town is located 738 km away from Addis Ababa, the capital of Ethiopia. There were a total of 60 registered CDROs actively serving the community in the town. Out of the 60 CDROs, 35 were drug stores, 23 were pharmacies and 2 were rural drug vendors. In Ethiopia, pharmacy, drug store and rural drug vendor differ in the range of medications allowed to stock or dispense and qualification of the professionals who run the retail outlets. Pharmacy is run by a licensed pharmacist with a minimum qualification of bachelor degree in pharmacy, drug store is run by licensed druggist or pharmacy technician with a minimum qualification of diploma in pharmacy and rural drug vendor is run by licensed health assistant with a qualification of certificate. The data for the study were collected from August 3, 2020 to September 21, 2020.

### Study design

A descriptive cross-sectional study design was conducted. A simulated-client (SC) method of visit was implemented to collect data. SC method can be considered as a robust methodological tool for pharmacy practice research especially if knowing being observed leads to behavioral modification [21–23]. For simulated evaluation of CPPs’ patient assessment and response to symptoms, a pre-prepared diarrheal patient scenario was used so that SCs answer queries accordingly (Table 1). The scenario was developed based on the common mnemonic, WWHAM which is used for patient interview during self-medication in community pharmacy practice [24], and the WHO recommendation for assessment and treatment of a diarrheal patient [25, 26]. WWHAM stands for W, who is the patient?; W, what are the symptoms?; H, how long have the symptoms been present?; A, actions?, has something already been done to stop the problem?; and M, medicines?, does the patient currently take any medicines for other problems? [24]. The scenario was designed to come up with an empirical diagnosis of bloody diarrhea (dysentery). The right decision by a CPP is to dispense oral rehydration salt (ORS) and advise the client to immediately take the patient to a health facility.

**Table 1:** Diarrhea patient scenario for SC visit, Gondar town, north-west Ethiopia, August 2020

SC request on entering a CDRO	Additional information to be provided by SC when asked by a CPP
I need medication for diarrhea	For whom: my wife (female) Age: 28 years Pregnancy status: 2 months pregnant Appearance of diarrhea: bloody and mucoid Onset and duration of diarrhea: since last day Frequency and amount of diarrhea: not quite sure but has occurred several times up to now Additional symptoms: had vomiting, fever, abdominal cramp and tenesmus Any triggering factor (pre-illness practice): had meal at restaurant where she is working a day before Any other health problem: no Action taken: nothing Medication for the illness or other purpose: not at all

### Population

All CDROs in Gondar town were the study population.

### Data collection procedure

Data collection tool was prepared in line with the SP scenario. The general categories of patient history taking questions were according to WWHAM mnemonic [24] and specific questions were developed based on the WHO recommendation for assessment and treatment of a diarrheal patient [25, 26]. A pretest on the data collection tool was done on 10 CDROs in Bahirdar town. After the pretest, information under assessment and response to a diarrheal case was modified to be detailed enough. The data collection was done by two pharmacy technicians. The pharmacy technicians were new professionals who were recruited while they process their licensure from Amhara Regional Health Bureau in Bahirdar town. Half-day discussion and rehearsal was done on the tool and process of data collection before starting gathering data. The principal investigator guided the data collectors on how to act as a SC. During the training, repetitive role-plays were performed by the data collectors acting as a CPP and SC interchangeably with the one playing SC role filled the data collection tool at the end. After each role-play, the data collectors gave peer comments to each other and the principal investigator evaluated the whole process and forwarded feedbacks. The rehearsal session was completed after making sure that the SCs were capable enough to perform the acting. During the actual data collection, to minimize recall bias, both SCs entered the CDROs together, one of them conversing with the CPP and the other critically following the process. They approached the dispensers as lay individuals by avoiding medical words and using lay community terms instead. If the dispenser was not willing to issue medication, the data collectors refrained from pressurizing and hence walked away. The data collectors provided information only when asked by the dispenser. They recorded what happened in the interaction on the data collection tool together immediately after the visit by making sure that they are out of sight. Confusions and problems faced during the actual data collection were discussed together and strategies were devised for the next visits. The data collected each day were checked for omissions, incomplete answers and illegible writing, and any problem encountered was corrected.

### Data entry, analysis and interpretation

The data were entered in epi info version 5 and analyzed using SPSS version 20. Descriptive statistics was done. Proportions were used to summarize the data and table and figure were used to present the findings.

### Ethical considerations

Ethical approval to conduct the study was obtained from the ethical review committee of School of Pharmacy, University of Gondar (Reference Number—SoP/811/12). The confidentiality of data obtained was assured starting from the design of the data collection tool by making sure that no direct dispensary identifier information was gathered. Filled data collection tools were kept secured by the investigators.

## Results

The response rate was 100% as data were collected from all 60 CDROs which were actively working during the study period.

### History taking

All of the 60 CDROs consulted for diarrhea asked at least one question. Age of the patient 59 (98.3%), whether diarrhea is watery or bloody 55 (91.7%) and onset and duration of diarrhea 46 (76.7%) were the three most commonly requested questions. No dispensary asked past-medical history, if the patient was taking medication for other purposes and whether the patient had tried any medication for the existing complication. Pregnancy status and pre-illness feeding practice were enquired in 5 (8.3%) and 18 (30%) of the CDROs, respectively (Table 2).

**Table 2:** Questions asked by providers in response to an adult diarrheal case in CDROs in Gondar town, north-west Ethiopia, August 2020 (*N* = 60)

History taking questions	Number of providers who asked	
	N	%
Age of the patient	59	98.3
Sex of the patient	12	20.0
Pregnancy status	5	8.3
Onset and duration of diarrhea	46	76.7
Frequency and amount of diarrhea	35	58.3
Whether the diarrhea is watery or bloody	55	91.7
Whether the diarrhea is mucoid or not	32	53.3
Presence of tenesmus	21	35.0
Presence of fever	31	51.7
Presence and site of abdominal cramp	24	40.0
Presence of vomiting	19	31.7
Past-medical history	0	0.0
Pre-illness feeding practice	18	30.0
Whether the patient is taking medication for other purpose	0	0.0
Whether the patient tried any medication to relief from the existing complication	0	0.0

### Outcome of visit

Medication was dispensed in 57 (95%) of the visits. Three of the consultations were followed by direct referral to a health facility to be seen by a practitioner as soon as possible without dispensing any medication. All of the immediate referrals were from pharmacies in which dispensers know the case involves a pregnant woman. Dispensing ORS and direct referral was recommended by 3 (5%) of dispensaries. No recommendation to be checked by practitioner was given in 54 (90%) of the visits (Table 3).

**Table 3:** Outcome of visit for an adult diarrheal case in CDROs in Gondar town, north-west Ethiopia, August 2020 (*N* = 60)

Outcome of visit	Number of CDROs	
	N	%
Referred/advised to be checked by a practitioner as soon as possible without dispensing a medication	3	5.0
Dispensed medication for rehydration only and referred/advised to be checked by a practitioner as soon as possible	3	5.0
Dispensed medication other than or in addition to drug for rehydration and no recommendation to be checked by a practitioner given	54	90.0
Total	60	100.0

### Medications dispensed

**Fig. 1:**
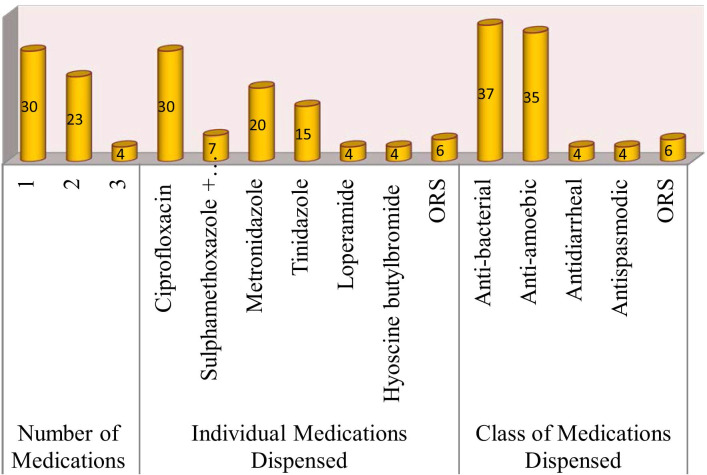
Medications dispensed for an adult diarrheal case in CDROs in Gondar town, north-west Ethiopia, August 2020 (*N* = 57)

**Fig. 2:**
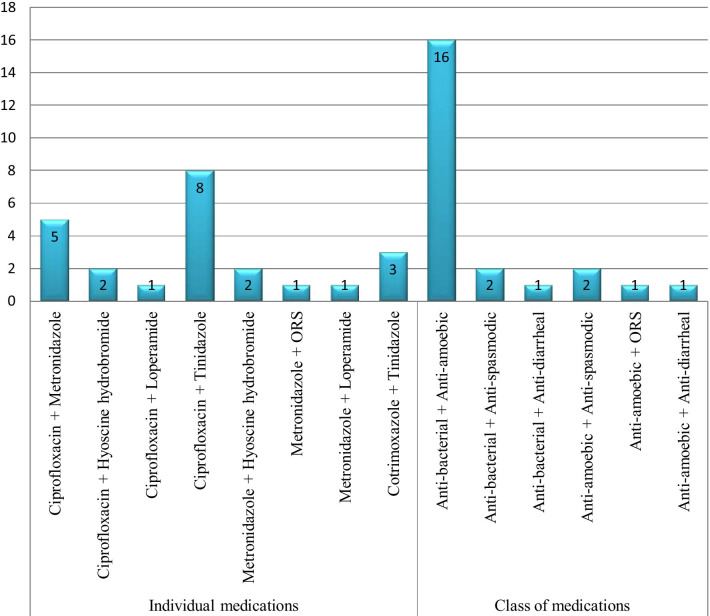
Two-medication combinations dispensed for an adult diarrheal case in CDROs in Gondar town, north-west Ethiopia, August 2020 (*N* = 23)

Among CDROs where drug was dispensed, a single drug was used in 30 (52.6%), two drugs were used in 23 (40.4%) and three drugs were dispensed in 4 (7.0%) cases. Overall, the most commonly recommended medications were ciprofloxacin 30 (52.6%) and metronidazole 20 (35.1%). When the pharmacotherapeutic class of medications is considered, anti-bacterials were issued in 37 (64.9%) occasions followed by anti-amoebics with 35 (61.4%). ORS was considered in only 6 (10.5%) dispensaries. After requesting and confirming pregnancy, ciprofloxacin and metronidazole were issued in one case (Fig. 1). Of the two drug combinations, ciprofloxacin + tinidazole was used in 8 (34.8%) visits and anti-bacterial + anti-amoebic was the most frequent medication-class combination dispensed with 16 (69.6%) (Fig. 2).

## Discussion

This study assessed the practice of responding to symptoms among CDROs in Gondar town, north-west Ethiopia, using an adult diarrheal patient scenario. The most frequent questions requested in our study were age of the patient (98.3%), whether diarrhea is watery or bloody (91.7%), and onset and duration of diarrhea (76.7%). However, a study in Iraq which was based on a middle-aged female diarrheal case documented number of episodes of diarrhea (82.7%), duration of diarrhea (78.7%) and presence of diseases and drug taken (77.3%) were the most common enquires by the surveyed pharmacies [27]. With regard to age of patient, other studies in Ethiopia also reported a higher proportion of inquiry for age by CPPs with 90.3% [16] and 98.3% [17]. The other issue of concern is past-medical history and medication history. Surprisingly, no one asked past-medical history, whether the patient was taking medication for other purposes as well as for the existing condition. This is in agreement with the study conducted in Pakistan which showed that only rarely (7.8%) did the pharmacy personnel ask about history of medication use [11]. Ayele et al. (2018), in their study on management of minor ailments including diarrhea in a community pharmacy setting in Gondar town, also found queries about past-medical and medication history by CPPs were insignificant [18]. Another study by Mengistu et al. (2019) in two towns of eastern Ethiopia documented none of the pharmacy professionals asked about medication history [17]. This indicates pharmacy personnel are less interested in medication history and they hardly relate symptoms with drug adverse effects, whereas it should have been their major role. Fever is highly important to differentiate the type of diarrhea whether it is because of an infectious agent or not. Unfortunately, only half (51.7%) of the dispensaries asked about the presence of this symptom. Even though pregnancy status is the most sensitive issue in drug selection for women in child bearing age, 91.7% of the CDROs we surveyed failed to ask this. Additionally, pre-illness feeding practice should be asked to identify any triggering factor for diarrhea. But, only 18 (30%) dispensaries do so. This is contrary to a study in Iraq in which 64% of community pharmacists inquired about pre-illness practice [27]. Overall, the degree of history taking in our study is unsatisfactory.

It is revealed that 95.0% of dispensaries in this study handed out medication and referral to a health facility is a rare practice. The extent of dispensing medication is quite higher than in other studies. Hussain and Ibrahim (2012) found medication was dispensed in 77.1% of the visits and only a few pharmacies choose referral of patients for medical evaluation [11]. Another study in Gondar town indicated 86% of simulated visits for acute childhood diarrhea and upper respiratory infection were provided with one or more medication and only 10.6% of CPPs advised simulated patients to visit a physician [19]. Other international studies also indicated community pharmacies prefer to dispense medications as all of them issued at least one medicine for every visit [12, 24, 27]. The right decision according to the scenario used in our study was to dispense ORS and recommend an immediate visit to health facility. This was practiced by only 3 (5.0%) of the providers.

In our study, the most commonly recommended medications were ciprofloxacin (52.6%) and metronidazole (35.1%). Anti-bacterials (64.9%) followed by anti-amoebics with 35 (61.4%) were the class of medications frequently utilized. Metronidazole was the most commonly given drug for diarrhea cases in a study in Saudi Arabia with a higher proportion (89%) [28]. Anti-amoebic drugs including metronidazole were commonly used (58.7%) in a study in Pakistan [11]. This is contrary to a study in Iraq where antidiarrheal drugs were used frequently [27]. ORS should be the mainstay of antidiarrheal treatment especially when fluid loss is high, but it was rarely used in our study. This is in congruence with a study in eastern Ethiopia where more than 90% of pharmacists answered they recommend ORS plus zinc when asked using a self-administered questionnaire, but only 13.3% of them actually dispensed it for a simulated patient [17]. A relatively higher use of ORS plus zinc preparation was observed for management of children’s acute diarrhea in five towns of Ethiopia with 29.6% [16] and much better use of ORS was observed in a Nigerian study [12]. The possible explanation for high utilization of anti-bacterials and anti-amoebics unlike ORS in this particular study is the scenario used which is according to bloody, mucoid and adulthood diarrhea. Among visits with two drugs dispensed, anti-bacterial + anti-amoebic was used in 69.6% of cases, which indicates dispensers are more likely uncertain whether a particular case is due to protozoal or bacterial cause and hence recommend both. However, this incurs unnecessary costs and side effects to a patient. The best decision in case of uncertainty should have been referring for further investigation. All the medications dispensed except ORS and hyoscine butylbromide are POMs in Ethiopia [29]. Several studies done in different parts of the world have also reported the non-prescribed sale of antibiotics by community pharmacies for a variety of ailments [30–34], which reaffirmed that such dispensing malpractice is a global problem and needs serious attention.

Overall, patient assessment practice in response to symptom-based presentation is far below expectation. A Low level of medication history taking is among the issues to mention as it should have been the main area of concern for pharmacy professionals. Similar studies in Ethiopia and other parts of the world also found such inadequacies in patient assessment [11, 12, 17, 35]. Insufficient patient assessment by pharmacists can be an indication of poor knowledge on one hand or reluctance to execute what they know in to practice. It ultimately leads to incorrect diagnosis and wrong decision as studies indicated the extent of questioning and information exchange between providers and clients determined appropriateness of outcome [36, 37]. The final response to the simulated diarrheal case in our study was also found to have different discrepancies. The appropriate outcome of visit according to the scenario used in our study is to dispense ORS and advise the client to immediately take the patient to a health facility. However, only three CPPs did so. Besides, only 10.5% of CPPs dispensed ORS and it is common to dispense POMs such as antimicrobials instead. Lack of adequate and continuous clinical training among CPPs is one important cited reason for the poor diagnosis and management of minor ailments [18]. But, having appropriate knowledge does not necessarily bring rational community pharmacy practice. It is evidenced by a significant difference in self-reported knowledge and actual practice of CPPs in the management of cases including non-prescribed dispensing of antibiotics rather than referral [12, 17]. One potential triggering factor for this is the financial interest of CPPs and owners. CPPs are forced to sell POMs without prescription to cope up with the financial competition and satisfy their customer demand or otherwise they will lose them to nearby CDROs [19, 34]. So, improving patient management by CDROs needs a wide range of interventions. Providing clinical training and implementing feedback mechanisms are important to equip professionals with the necessary knowledge and skill [18, 36, 38]. Strict regulatory control to monitor whether CDROs abide by OTC medicines is essential [19]. Compensation for service delivery [18] particularly payment for counseling without dispensing medication is relevant to recognize the professional service of CPPs, minimize the non-prescription sale of POMs, and ultimately changes the linkage of financial survival with sailing medicines. Upgrading community awareness on the extent of the role of CDROs also avoids unnecessary pressure from clients and increases trust [18].

The study is not without limitations. The finding of the study may not be generalizable to the whole CDROs in the country as it is confined to Gondar town. Despite privacy concern, it would have been better if an audio recording was done to avoid total reliance on the memory of SCs. Although SCs wrote their conversation on the data collection tool immediately after each visit, recall bias cannot be completely ruled out. The qualification of providers cannot be collected and even they may be non-professionals at times. The quality of counseling and advice given by the providers was not addressed. Even with these limitations, the present study informed gaps in managing adult diarrheal cases by CDROs which can be taken as a base for a comprehensive investigation of community pharmacy service in Ethiopia.

## Conclusion

The present study indicated history taking by CDROs for an adult diarrheal case is poor with key patient assessment questions for decision-making missed. Referral decision and the use of ORS is a rare practice. Majority of the dispensaries issued antimicrobials illegally. Educational interventions coupled with incentive mechanisms for cognitive pharmaceutical service and strict regulatory enforcement are needed to reduce the problem. A wider scope of research on community pharmacy practice is recommended.

## Data Availability

The datasets used and/or analyzed during the current study are available from the corresponding author on reasonable request.

## Abbreviations

CDRO: Community drug retail outlet
CPP: Community pharmacy professional
ORS: Oral rehydration salt
OTC: Over the counter
POM: Prescription-only medicine
SC: Simulated-client
